# Cell-type-specific densities in mouse somatosensory cortex derived from scRNA-seq and *in situ* RNA hybridization

**DOI:** 10.3389/fnana.2023.1118170

**Published:** 2023-03-02

**Authors:** Daniel Keller, Csaba Verasztó, Henry Markram

**Affiliations:** Blue Brain Project, École polytechnique fédérale de Lausanne (EPFL), Geneva, Switzerland

**Keywords:** cell density, mouse brain, somatosensory cortex, transcriptome, scRNA-seq, cell type

## Abstract

Cells in the mammalian cerebral cortex exhibit layer-dependent patterns in their distribution. Classical methods of determining cell type distributions typically employ a painstaking process of large-scale sampling and characterization of cellular composition. We found that by combining *in situ* hybridization (ISH) images with cell-type-specific transcriptomes, position-dependent cortical composition in P56 mouse could be estimated in the somatosensory cortex. The method uses ISH images from the Allen Institute for Brain Science. There are two novel aspects of the methodology. First, it is not necessary to select a subset of genes that are particular for a cell type of interest, nor is it necessary to only use ISH images with low variability among samples. Second, the method also compensated for differences in soma size and incompleteness of the transcriptomes. The soma size compensation is particularly important in order to obtain quantitative estimates since relying on bulk expression alone would overestimate the contribution of larger cells. Predicted distributions of broader classes of cell types agreed with literature distributions. The primary result is that there is a high degree of substructure in the distribution of transcriptomic types beyond the resolution of layers. Furthermore, transcriptomic cell types each exhibited characteristic soma size distributions. Results suggest that the method could also be employed to assign transcriptomic cell types to well-aligned image sets in the entire brain.

## 1 Introduction

Mapping the anatomical location of individual neuronal and other cell types within the cerebral cortex is key to understanding how the various cell types participate in brain circuits. This is because neuron subtypes exhibit different input-output transfer functions, electrical and morphological properties. Their geometrical position also influences how they are wired together in the cortical microcircuit. Advances in the measurement of single-cell transcriptomes are augmenting neuronal subtype classification, even though these classes have not yet been fully established. In this work, we show that well-aligned *in situ* hybridization images can be used to map transcriptome-defined cell classes with sub-layer specificity.

The cellular composition of a variety of tissues can be inferred based on the fact that cells express constellations of genes in a type-specific manner. For example, cellular localization in zebrafish embryos has been described by matching single-cell sequencing of total RNA (scRNA-seq) data with *in situ* RNA patterns (Satija et al., 2015). Combining mRNA profiles with a cell atlas has been used to assign cells to precise locations in the brain of the marine annelid *Platynereis dumerilii* (Achim et al., 2015).

Similar approaches for inferring composition have also been employed in the brain. In early work, transcriptome data was fitted to the Allen Brain Atlas data to obtain whole-brain predictions of cellular compositions for adult mouse (Grange et al., 2014). During the fitting process, they found that *in situ* hybridization (ISH) data exhibited large variability in quality, and they discarded genes that exhibited high variability. The method was also refined to identify the best genes for the fitting process (Mezias et al., 2022). Correlational techniques were used to link projection types to specific genes in ISH datasets (Sorensen et al., 2015). Reverse transcription polymerase chain reaction (RT-PCR) expression has been used to predict cellular composition in juvenile rat somatosensory cortex (Keller et al., 2019). Mouse brain cell type atlases have also been made using subsets of highly expressed gene markers from each cell type (Erö et al., 2018).

Advances in spatial transcriptomics technology allow more transcript information per cell to be collected within the same specimen (Lein et al., 2017). For example, fluorescent labeling of the same cells for a set of marker gene transcripts permits assignment of cell types (Codeluppi et al., 2018; Park et al., 2021). The use of 10x genomics, with quantification of many transcripts in the same spatial location, also has potential (Rodriques et al., 2019; Stickels et al., 2021). In the hippocampus, sequential fluorescence *in situ* hybridization (seqFISH) has been used to profile 249 genes at cellular resolution for the purpose of characterizing the spatial distribution of cell classes (Shah et al., 2016). Despite these advances in multiple transcript registration, often performed on single slices in a piecemeal manner, we cannot ignore that there remains a large amount of ISH data from the Allen Institute for Brain Science (AIBS) which comprehensively covers large swathes of the brain. Accordingly, it is still desirable to use such data for both cell placement and validation.

Transcriptomic mapping and classifying of brain cells based on scRNA-seq is making strides, but no definitive standard has yet emerged. Zeisel et al. (2018) developed a hierarchical, data-driven taxonomy with 265 clusters. Various brain regions have also been characterized, for example, the isocortex and hippocampal formation (Yao et al., 2021). Our understanding of what a cell type is continues to evolve (Zeng, 2022), and subtypes may even depend on brain state (Bugeon et al., 2022).

An additional trend relevant to detailed cell mapping is progress in image registration that allows improved mapping of images to the standardized atlases (Krepl et al., 2021; Wang et al., 2021). In anticipation of well-aligned data eventually becoming available, we wondered what type of output from the cell composition fitting process could be expected with well-aligned images. Would this allow extraction of features with high spatial resolution? Since well-aligned machine-annotated images were not available for the entire brain, we used well-aligned, manually annotated images.

In this work, we combined a recent cortical RNA-seq dataset from AIBS (Yao et al., 2021) with a manually aligned set of Allen Brain Atlas gene expression pattern images to obtain predictions of cell-type distributions in the somatosensory cortex of the adult mouse. The pipeline makes use of cell soma size to predict the distribution of soma sizes of the cell types and compensate for differences in intensity caused by the variation in cell soma size, a problem that earlier efforts have also tried to overcome (Erö et al., 2018; Rodarie et al., 2022).

## 2 Methods

The overall goal of the process is to obtain the distribution of transcriptomically defined cell types in the somatosensory cortex of adult mice, as a function of cortical depth. To this end, there are several steps: processing of ISH image data, normalization of transcriptomics data, linear algebra solution of densities, iterative refinement of the transcriptomic distributions, and final density adjustment. The code is available for download at: https://github.com/BlueBrain/distributor.

### 2.1 ISH image processing

**Figure 1:**
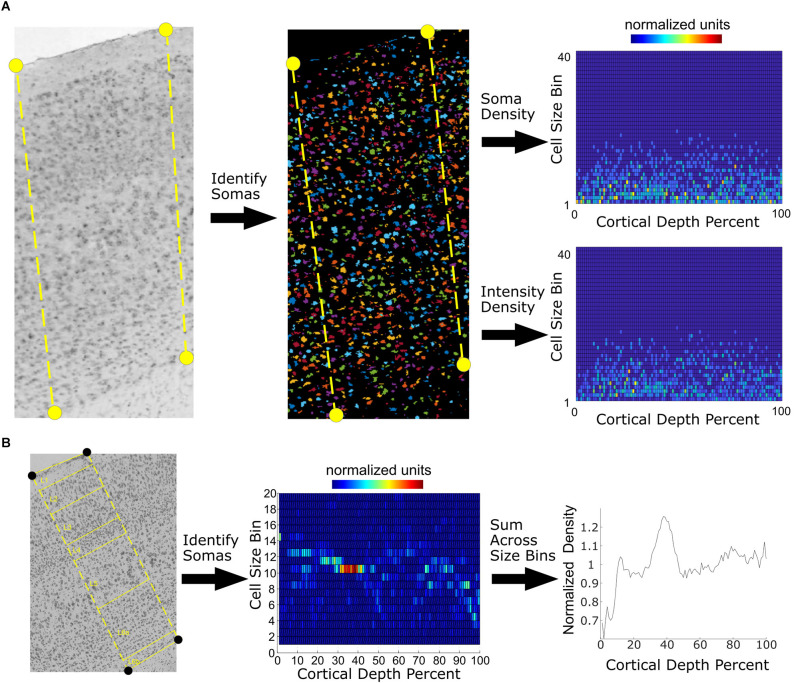
Counting of cell density and intensity as a function of cortical depth and size. **(A)** A region of somatosensory cortex, outlined by the yellow dashed lines, from L1 through L6 is manually annotated on *in situ* hybridization (ISH) slices. As an example, we take the image for 2510039O18Rik. Automatic detection of cell somas is performed on the image, and the background subtracted. Each identified spot is marked by a different color in the second image. Cell soma densities are mapped to 100 bins corresponding to their fractional depth. The top of the cortex corresponds to the first bin. The area of each cell body is also calculated, allowing further parcellation into 40 area-depth bins. The total intensity density as a function of area bin and cortical depth is also obtained. **(B)** Density obtained from Nissl slices. The densities are mapped to 100 depth bins and 40 size bins. Note that the intensity densities from Nissl slices are not used. By summing across the size bins, a plot of density vs. cortical depth percent is obtained.

The image data used in the process comes from the AIBS (Lein et al., 2007). This data was collected from male, 56-day-old C57BL/6J mice using *in situ* hybridization to label expressed RNA for most genes. Cell somas had the highest mRNA expression level. Nissl image data from the same source was also used. We manually annotated the contours of a cross-section of somatosensory cortex in the AIBS data (Figure 1) for 13,379 sagittal images and 1,454 coronal images. The annotation process marked the top of layer 1 and the bottom of layer 6. Noise in individual images can be significant due to the presence of air bubbles, fibers, folds, and tears that mar the image. Where possible, we avoided these regions of the image and omitted problematic images from further processing. Image processing was then performed on the cross-section in order to determine the soma positions and cell density as a function of cortical depth. We adhered to a convention in which 0% cortical depth corresponds to the top of layer 1 and 100% corresponds to the bottom of layer 6.

The image processing algorithm was implemented in Matlab (Mathworks, Natick, Massachusetts). The image processing algorithm first identified the baseline intensity of each image, taken as the most prevalent intensity in each annotated region. After subtraction of the baseline, circular regions of high intensity corresponding to putative cell somas were identified. In doing so, the algorithm measured the areas of the circular spots.

Since cell soma area varies according to the type of cell, we noted the area of the soma of each cell in normalized pixel units, with each pixel corresponding to an area of 1 μm^2^. Most cell soma sizes were under 200 pixels in area. We assigned the soma areas to bins, with each bin containing 20-pixel units, corresponding to a surface area of 22.9 μm^2^. There were 40 soma area bins in total and 100 cortical depth bins.

Soma density was taken as the total number of spots at a particular soma area bin divided by the total area of the slice at the corresponding cortical depth bin. This allowed creation of a two-dimensional map of the density as a function of both cell soma area and cortical depth. This was done for both Nissl and ISH images (Figure 1).

We also extracted intensity density from the ISH images. Intensity density was the total intensity of the somas at a particular soma area bin divided by the total area of the slice at a particular cortical depth bin. The intensity density was contained in the expression matrix E, which had the following dimensions: number of genes by 100 cortical depth bins by 40 soma area bins.

Two types of artifacts emerged during image processing. First, at the lower end of the size range, the algorithm identified large cellular processes such as dendrites in addition to cell somas. This sort of artifact was difficult to prevent. Second, we observed that brain slices occasionally fold and occlude layer 1. Therefore, predictions for layer 1 can be expected to be less accurate than predictions for other layers.

**Figure 2:**
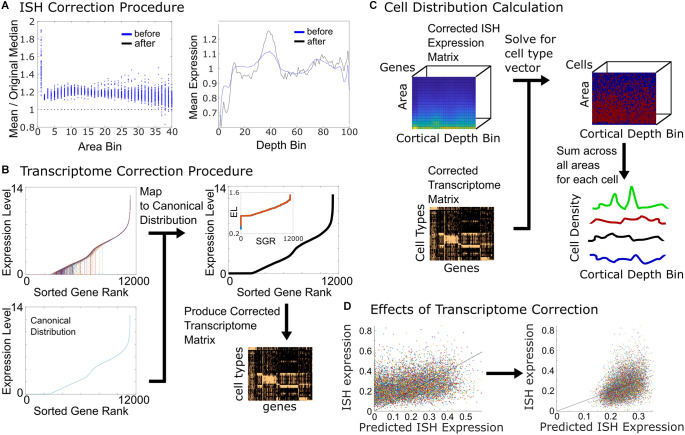
Correction procedures and cell density calculation. **(A)** Correction of image expression intensities is done on each bin by dividing intensity densities by the median of the expression, for all non-zero gene entries (left panel). Points in each area-depth bin before (blue) and after (black) normalization. In the right panel, the profile of the mean ISH expression as a function of cortical depth (blue) is also scaled to match the Nissl density profile (the black trace shows mean expression after rescaling). **(B)** Correction of cell-type transcriptomes. Plotting the sorted transcriptomes for each of the cell types (different colors) shows that they have different profiles and degrees of completeness. We wish to map them to the same canonical distribution (lower left panel, blue line). After mapping, all sorted transcriptomes have the same shape (black trace, upper right panel). The inset shows the final expression level vs. sorted gene rank, starting from two different seeds (red and blue). They converge to the same distribution. Adjusted transcriptomes are used to generate a corrected transcriptome matrix. **(C)** The corrected expression and transcriptome matrices are used to solve for the cell density in each area-depth bin. By summing across the area bins a cell density vector as a function of depth is produced for every cell type. **(D)** If an uncorrected transcriptome is used, the predicted ISH expression deviates substantially from the actual ISH expression (left panel). After optimization, the predicted vs. actual expressions better correspond (right panel). A line with unity slope is shown for reference.

Two normalization steps were performed. Within each area-depth bin, we applied a linear scaling factor to normalize by dividing its value by the median intensity density in each bin (Figure 2A). Furthermore, along the cortical depth, we applied a scaling factor to each cortical depth bin to make the mean profile of the intensity density match the Nissl density profile shape (Figure 2A).

### 2.2 Transcriptome normalization

Transcriptomes and their associated cell classification scheme were taken from a publicly available single-cell transcriptome dataset obtained from dissociated cells (Yao et al., 2021). This dataset provided a comprehensive repertoire of excitatory and inhibitory neuron types from the cortex. The naming convention used in this dataset used the character “x” followed by a number and finally a short descriptive string of the cell type.

Each of the cell types expresses different subsets of genes. We used the median values of the samples in this dataset to calculate a transcriptome matrix T, of dimensions number of genes by number of cell types. The transcriptome set was restricted to the cell types known to be present in cortex. There were 316 cell types in the regions of interest and 11,344 genes used after consolidation of multiple datasets in the original annotated images.

For each cell type, we plotted the sorted values of the transcriptomes (Figure 2B). The transcriptomes vary according to both their completeness as well as the shapes of their sorted value curves. In order to standardize the distributions, we mapped the sorted values of each cell type transcriptome to the same canonical distribution. We used as a starting point for the canonical distribution—the sorted transcriptome—of the most complete cell type of all the available transcriptomes.

The zero values of the transcriptome matrix for each cell type class were assigned a sorted rank order corresponding to the rank order of the average non-zero entries for that cell class. This was done in a sequential fashion, such that the finest cell classes were used to fill in zero values first. The fine classes are SST, PV, Lamp, VIP, and SNCG, as well as excitatory neurons in L2, L3, L23, L4, L5, L45, L6, and L6b. If there were still zero values because the finer class itself lacked an entry for particular genes, then a coarser class was used to fill in the ranks of remaining zero values. The next level of coarser classes was excitatory and inhibitory. After that, the next level of classes was neurons, astrocytes, vascular leptomeningeal cells (VLMC), oligodendrocytes, microglia, and smooth muscle cells (SMC). The final level of classes was all cells, which effectively filled in the rank of any remaining zeros. By this process, genes missing from any transcriptome could be assigned ranks from the closest possible cell class for which data was available.

### 2.3 Linear algebra solution

With the ISH expression matrix and the transcriptome matrix, we can solve the cellular composition within each of the area-depth bins (Figure 2C). The ISH expression level vector E_jk_ is assumed to be the product of the transcriptome matrix T and a cellular composition vector C*_jk_*:


(1) $$E_{ij}=T*C_{jk}$$

In the above equation j is the depth bins index and k is the area bin index. The equation can be solved for the cell-type-composition vector C*_jk_*:


(2) $$C_{jk}=T^{-1}E_{jk}$$

The non-negative least squares method in the Matlab toolbox allows numerical solution in each depth-area bin.

After the composition was solved for, in each bin a predicted ISH vector E*_predicted_* was calculated by multiplying the transcriptome matrix by the solved-for predicted cell density vector C*_jk_*. The predicted ISH expression could then be compared to the original ISH expression for each gene. A least-squares error was calculated to assess how well the original ISH expression could be matched. The square of the total error was the sum of the squares of the individual gene errors:


(3) $$Error=\sqrt{\sum_{i\in genes}^{}{\left(E_{predited\;i}-E_{vec\;i}\right)}^{2}}$$

We plotted the predicted summed expression for each gene vs. the original ISH expression. From this assessment, it became clear that some slices in the original experiments are more strongly or weakly labeled than might be expected from the prediction; hence there is scatter with respect to a straight line (Figure 2D, left panel).

### 2.4 Iterative refinement of transcriptome distributions

In order to make the predicted summed expression for each gene better match the ISH expression, we adjusted the canonical distribution and repeated the process of calculating the predicted expression and least-squares error of the prediction. The canonical distribution was modified by adding to the sorted values random values drawn from a normal distribution with variance 1, multiplied by a scaling constant. At each iteration, the canonical distribution was re-sorted in order to be monotonically increasing.

During each iteration of the process, canonical distribution alterations that reduced the total error were kept in the optimization process, which was run until convergence was achieved. Convergence was defined as no change in error value in 100 iterations. This typically took 10,000 iteration cycles. We found that the same solution was arrived at regardless of the initial mapping (inset of upper right panel, Figure 2B). The outputs of the optimization process were the canonical ranked distribution and the zero-value rankings, used to make a corrected genes matrix. After optimization, there was better agreement between the predicted ISH expression and the actual expression, though some differences remained (Figure 2D, right panel).

For non-neuronal cells, the final predicted value of the above process underestimated astrocyte percent composition. In order to adjust the non-neuronal cell percent composition within the framework of the above process to better match literature percentages (Gabbott and Stewart, 1987), we optimized the rank order of the zero values of genes with rank above a class-specific rank for each class to minimize the root-mean-square error of the predicted gene expression for those genes. The classes for which this was applied were astrocytes, oligodendrocytes, and microglia. Given that the number of non-zero entries varies widely among these classes, we believe this is a relatively benign way to allow literature data to influence the process.

### 2.5 Density adjustment

The corrected genes and ISH expression matrices were used in solving for the final cell composition in each area and depth bin (Figure 2C, bottom right panel). To obtain the distribution as a function of depth alone, the area bins were summed up in each depth position.

In order to anchor the normalized densities, a scaling factor was applied to all calculated densities in layer 2 through layer 6 so that the average neuron density in these layers would be 120,000 cells/mm^3^, in accordance with literature values (Keller et al., 2018). With the cell-distribution solution computed, the average soma-size-distribution kernels per cell type could also be computed by summing across bins of the same cell size.

## 3 Results

### 3.1 Excitatory types

**Figure 3:**
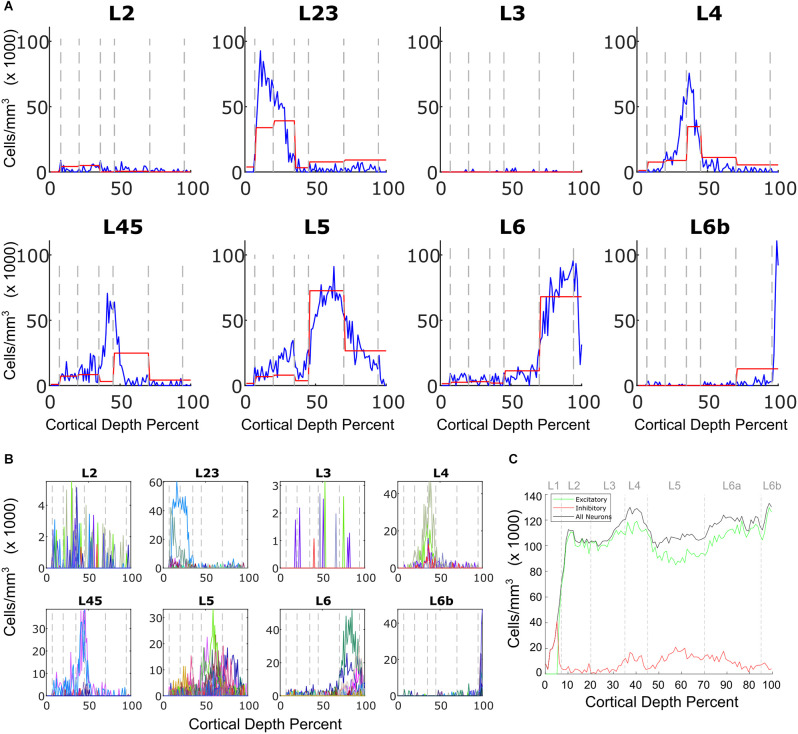
Excitatory neuron predictions. **(A)** Predicted cell densities for broad classes of excitatory cell types. On the x axis, 0 denotes the top of layer 1 while 100 is the bottom of layer 6. Cell density units are cells/mm^3^. Cell types for the most part are present where the transcriptome cell-type labels suggest they should be. The red line indicates the distribution of samples in the AIBS data. **(B)** A breakdown distribution of the subtypes whose sum is shown in **(A)**. Each color represents a different transcriptomic type. Since L2 and L3 cells were present at very low levels, there is much noise in their predicted distributions. **(C)** Excitatory (green) and inhibitory (red) profiles are shown, and total neurons (black). The inhibitory cell density reaches a peak in the superficial layers. In all graphs, layer boundaries for L1, L2, L3, L4, L5, L6a, and L6b are indicated by the dashed gray lines and placed at 8, 20, 35, 45, 70, 95, and 100 percent of cortical thickness, respectively.

Figure 3A shows the results of fitting of excitatory cell types for the P55 mouse, summed over layer-dependent categories. Most excitatory cells are present only in specific layers. In most layers they were placed in a tight configuration, though L2 and L3 pyramidal cells could not be effectively placed. L45 cells mostly appeared in layer 4, though experimentally most samples were obtained from layer 5. Figure 3B shows the breakdown of excitatory neurons into transcriptomically defined subtypes. These are present in different proportions and distributions. Even within the broader classes, rich substructure can be observed even within layers. For example, one type of L23 cell localizes to layer 2 (Figure 3B, L23 panel, x171_L23ITCTX). As another example, two types of layer 6 cells (x289_L6CTCTX and x288_L6CTCTX) localize to the lower part of layer 6.

The summed excitatory and inhibitory profiles are shown in Figure 3C, and exhibit features well-characterized in a review of the literature (Keller et al., 2018). Excitatory neurons have peaks in layers 2, 4, and 6, with the peak in layer 4 being particularly prominent. There is also a peak in layer 4, which reflects the thin strip of excitatory neurons characteristic of this layer. Inhibitory neuron density experimentally exhibits a peak at the layer 1 to layer 2 boundary, which is also observed in the prediction.

### 3.2 Mapping of interneuron types

**Figure 4:**
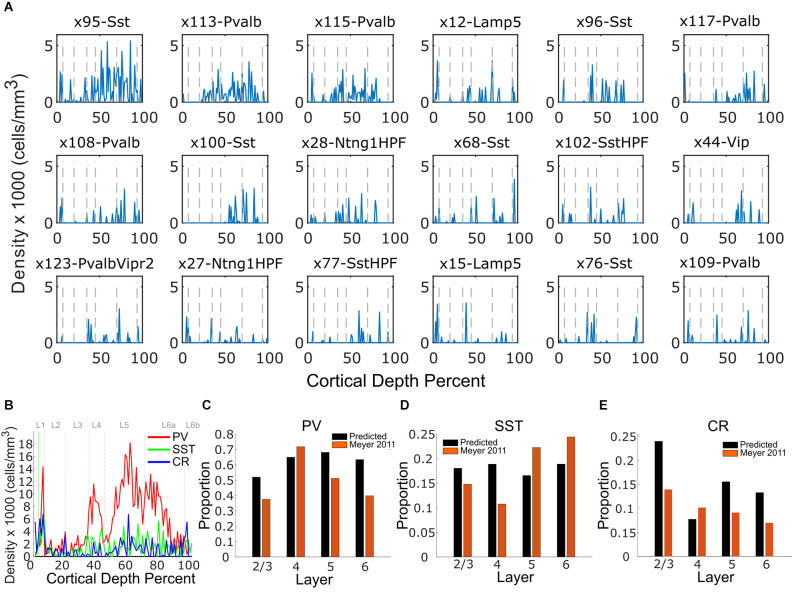
Inhibitory neuron predictions. **(A)** Predicted expression of inhibitory cell types as a function of percent cortex depth (blue traces), shown for the most frequent interneuron types. **(B)** Average density of parvalbumin (PV; red), somatostatin (SST; green), and calretinin (CR; blue) expressing cells. **(C)** Layer-dependent inhibitory composition comparison against the literature (Meyer et al., 2011) for PV. **(D)** Comparison for SST. **(E)** Comparison for CR. In all density plots, layer boundaries for L1, L2, L3, L4, L5, L6a, and L6b are indicated by the dashed gray lines.

Interneurons are highly diverse, with numerous types. The algorithm placed the top three types, x95-Sst, x113-Pvalb, and x115Pvalb with largely contiguous distributions (Figure 4A). Due to the high number of subtypes relative to total inhibitory densities, noise was present in the predicted distributions of the rest (Figure 4A). Nonetheless, for broader classes of summed interneurons (Figure 4B) the results were comparable to the literature (Figures 4C–E).

We compared the layer-wise distributions against literature measurements of somatostatin (SST), parvalbumin (PV), and calretinin (CR) expression in Figures 4C–E. Parvalbumin-expressing interneurons are experimentally more predominant than SST-expressing interneurons. Furthermore, parvalbumin is not as expressed in L23 as it is in the deep layers, consistent with experimental observation (Inzunza et al., 2003). CR-expressing cells are present in the lowest density of the three markers studied. Consistent with experiment, CR-expressing cells are highest in L23.

### 3.3 Mapping of other cell types

**Figure 5:**
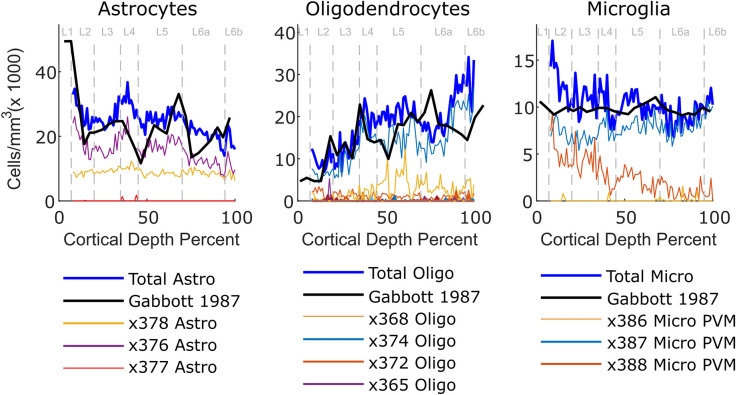
Assignment of single cells to cortical positions based upon their transcriptomes for astrocytes, oligodendrocytes, and microglia. The thick dark blue shows the summed predicted traces, while the thinner colored traces are the subtypes of each predicted class. The density in layer 1 was quite high and likely artifactual, so is omitted in order to better show the features of other layers. The thick black traces in the top rows show experimental densities from Gabbott and Stewart (1987); for the rat. In all graphs, layer boundaries for L1, L2, L3, L4, L5, L6a, and L6b are indicated by the dashed gray lines.

Non-neuronal cell distributions were also predicted (Figure 5). They exhibited both similarities and differences with experimental measurements. The Gabbott and Stewart comparison experiment is one of the few in the literature with quantitative density data, but it is from rat visual cortex (Gabbott and Stewart, 1987). This might partially explain divergent aspects of the comparison; for example, the microglia prediction had higher density in superficial layers, in contrast to the experimental measurement. Nonetheless, the total oligodendrocyte density was higher in deep layers, in agreement with experimental observations (Gabbott and Stewart, 1987; McGee et al., 2005). Furthermore, the combined predicted astrocyte density exhibited peaks at the same places as seen in experiment. Endothelial, VLMC, and smooth muscle cells were not well placed by the algorithm, possibly because they are not well stained or because the image processing algorithm does not extract them.

### 3.4 Size kernels

**Figure 6:**
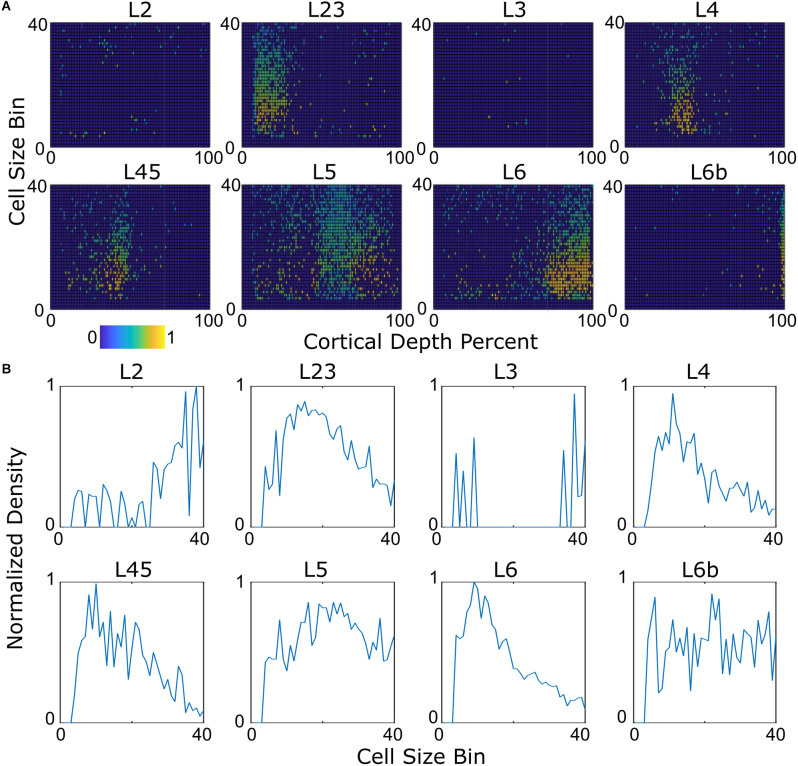
Characteristic size distributions. **(A)** The distribution plotted in area-depth bins. L2 and L3 cells do not have a coherent pattern, indicating that the method may not correctly place them. **(B)** Average size distribution kernels. Differences in the kernels are observed. L4 cells tend to be slightly smaller than L5 cells, for example.

Presumably, cells of a given type will exhibit the same distribution of soma sizes no matter where in the cortex they are located. Figure 6A shows example profiles for excitatory types, and Figure 6B shows the average values as a function of cell size bin.

Some cell kernels, such as pyramidal cells in layer 6b, exhibit more than one peak (Figure 6B). This may reflect subtype heterogeneity or cell bodies whose overlap meant that they could not be split into two separate cells.

## 4 Discussion

The distribution of excitatory neurons behaves largely as expected. Pyramidal cells classified as belonging to a particular layer largely appear in that layer. The results place several subpopulations of excitatory neurons in each layer, consistent with reports that different projection classes of neurons are present (Sorensen et al., 2015).

For cells present at lower densities, such as inhibitory interneurons, more discord between the results of the depth-bin technique vs. the method of using both the depth and size bins is seen. Overall, the percent of neurons that are inhibitory is 11.5% in rat somatosensory cortex (Meyer et al., 2011). The measurement in mouse cortex is 12% (Loomba et al., 2022). The prediction from this work is 10.9%. When the total densities of SST- and PV-expressing cells are plotted, the profiles look similar to what is observed experimentally. We attribute differences to noise in the images due to low numbers of replicates, and to the fact that some cell types may not be represented in the input transcriptome dataset.

The method relies on several assumptions. We assume that the adjusted transcriptome expression level of a gene and the normalized ISH intensity in the same cell can be linearly related. Modeling nonlinear effects would be challenging. We also did not explicitly compensate for stereological effects caused by the cutting of cell somas in the slicing process. The method also assumes that the Nissl stain captures all cell types and that all cell types exhibit the same canonical shape of ranked gene expression. Furthermore, we do not account for cell occlusion effects. Finally, we also assume that the classification of cells is complete, but in fact, additional subtypes probably exist. Including these categories when they become better defined would result in better fitting.

The goal was not to accurately determine transcriptomic state, which would require quantitative PCR experiments, but rather to find the best fit of cell transcriptomic states to the ISH data. Even though any one particular ISH signal might have a certain degree of noise, using thousands of markers allows even faint patterns to show up. Due to the large number of genes the effect of error in any particular gene has a small effect.

The input data is of relatively high quality. The large number of cellular stains were created with the same automatized protocol with strict environmental control (see http://help.brain-map.org/display/mousebrain/Documentation). Furthermore, we relied on the Allen Mouse Brain Atlas project’s own densitometric analysis (across reciprocal negative and positive control sections, as well as across multiple experimental runs), which qualitatively scored each ISH runs and discarded outliers to maintain consistency. While we had no way to influence or quantify the intrinsic experimental (ISH probe penetration speed, cell section level/depth in tissue, non-specificity), run-to-run (independent riboprobe synthesis, ISH probe quality and size), and biological variability (tissue permeability, genetic variability) influence upon expression levels and quality, we could overcome most of the effects by scaling the image data and correcting for nonspecific staining. Nevertheless, our analysis only used the detected relative mRNA expression levels to draw distinctions between cells, and these values were considered as what they were (as points on an affine line). We are aware of the limitations of this analysis, that in extreme cases even non-linear relationships between expected gene expression values and pixel intensity can break down. While we think our method can be legitimately used to make comparisons with populations of cells, we consider the independent validation of our data as a viable future avenue in our research.

This effort advances on previous efforts in that it seeks to assess what can be expected from a high-resolution alignment, and what can be expected once machine-learning approaches provide this. Transcriptome-based cell density prediction may be easier to apply to the whole brain than large-scale sampling would be. If only fitting of raw intensity data is used, the technique scales well with data size since the optimization problem needs to be solved on a per layer/bin basis, not on the entire brain.

The somatosensory cortex example used in this work relied on some averaging of expression density at the same cortical depth. Consequentially, one might wonder if it would translate to other brain structures that lack a layer structure. We believe that in such cases it would be possible to develop local averaging kernels if averaging is needed, but that this might not be necessary if the number of cell types in non-laminar structures is lower.

A novel feature of this work is that we extracted the characteristic cell soma size profiles, and corrected artifacts arising from differences in cell soma size. Soma size information may have additional use in cellular feature assignment. Indeed, further fitting could be performed under the constraint that cells of a given type must adhere to the same size distribution kernel, but we did not attempt to do so. This remains a topic for future research.

Already, efforts to use transcriptomes published in the literature with the Allen Brain Atlas gene expression data have resulted in considerable success (Grange et al., 2014). We build upon this work in order to solve for cortical cell distributions at a high level of spatial detail. Broad qualitative agreement in cell densities is observed using this fitting technique. Full quantitative agreement will require filling in gaps in cell transcriptome profiles through the collection of more examples and the assignment of additional sub-types. We see no conceptual roadblocks standing between the acquisition of improved data and the generation of type-specific neuronal circuits suitable for simulation.

## Data availability statement

Publicly available datasets were analyzed in this study. This data can be found here: https://mouse.brain-map.org/.

## Author contributions

DK conceived the study. CV and HM provided scientific feedback. All authors contributed to the article and approved the submitted version.
